# Cobb’s Tufts: A Systematic Review

**DOI:** 10.7759/cureus.20151

**Published:** 2021-12-04

**Authors:** Ibrahim Almafreji, Alex Manton, Fraser S Peck

**Affiliations:** 1 Emergency Department, University Hospital Hairmyres, East Kilbride, GBR; 2 Cardiology, Christchurch Hospital, Christchurch, NZL; 3 Pharmaceutical Medicine, Richmond Pharmacology, London, GBR; 4 Ophthalmology, Eastbourne District General Hospital, Eastbourne, GBR

**Keywords:** hyphema, spontaneous hyphema, iris microhaemangioma, iris vascular tufts, cobb's tufts

## Abstract

Cobb's tufts, also known as iris vascular tufts (IVT) and iris microhemangiomas (IMH), are coils of tightly clustered, minute blood vessels at the iris pupillary border. This study aimed to analyze previous literature and provide an update on Cobb’s tufts. A systematic literature review was carried out by interrogating PubMed, Google Scholar, Cochrane, and Embase databases. Full-text English language articles of any year were included in this study. A total of 38 articles fulfilled our inclusion criteria. A total of 115 reported cases of Cobb’s tufts were incorporated into our review. The age of the patients ranged between 36 and 86 years. No sex or racial predisposition was noted. Most patients had no history of trauma, surgery, or blood dyscrasia. The majority of cases are asymptomatic and bilateral unless a spontaneous hyphema occurs, which most commonly presents as blurred vision. The etiology of this condition remains uncertain; however, a higher incidence has been shown in systemic conditions such as myotonic dystrophy and diabetes. Fluorescein angiography can be utilized to investigate tufts. Management includes treatment of raised intraocular pressure, observation for single bleeds, laser therapy for recurrent hyphemas, and lastly, iridectomy, which is considered in cases of recurrence following laser treatment.

## Introduction and background

Cobb's tufts, also known as iris vascular tufts (IVT) and iris microhemangiomas (IMH), are true hamartomas of the iris stromal blood vessels [1]. The first case of a spontaneous hyphema with iris microhemangioma was reported by Tyson in 1932, according to Fechner’s article in 1958 [2]. In 1969, Cobb provided an observational study providing a detailed description of the condition [3]. This led to them being eponymously named Cobb’s tufts. Cobb noted that these lesions protruded forward from the iris in single or multiple loops, were vascularized, adjacent to pupillary ruff, and were separate from each other. In a later publication, he also associated the occurrence of vascular tufts in patients with myotonic dystrophy and diabetes [4]. They can uncommonly present as spontaneous hyphemas, which can lead to high intraocular pressure (IOP) and may potentially cause irreversible damage to the optic nerve [5]. There is a lack of good quality evidence as to the management of this condition. To our knowledge, the last review of the literature was in 2013 [6]. The purpose of this study was to analyze current literature and provide a comprehensive update on all aspects of this condition.

## Review

Methods

We present a systematic review of the literature on Cobb’s tufts following Preferred Reporting Items for Systematic Reviews and Meta-Analyses (PRISMA) guidelines [7]. The PRISMA statement and checklist were used to critically analyze articles and structure this review [7]. Our literature search extends from 1958 to February 2020. The systematic search was conducted through PubMed, Embase, Google Scholar, Cochrane, and the reference lists of the articles. We utilized the following terms in our search: Cobb’s tufts, iris microhemangiomas, iris vascular tufts, and neovascular tufts.

**Figure 1:**
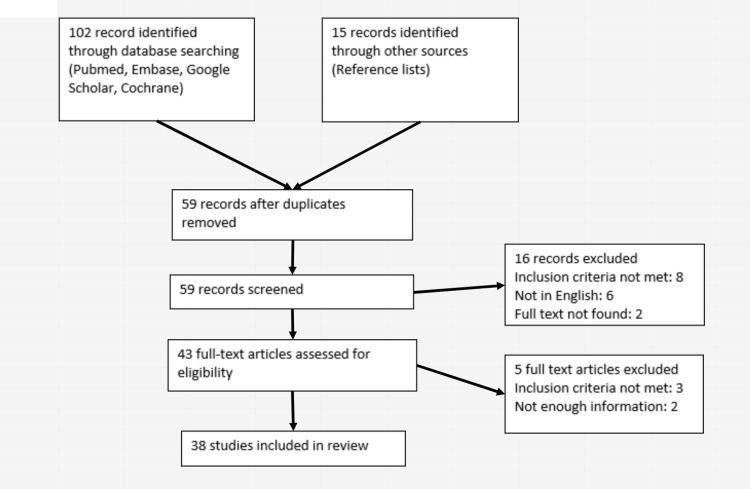
Flowchart outlining the systematic review process.

Studies published from any year in the English language were included. Studies that reported spontaneous hyphemas without evidence of IVT and iris neovascularization (rubeosis iridis) were excluded. Moreover, editorials and correspondences were also included. The study identification and selection process are demonstrated in Figure 1 [7].

Full texts were obtained for articles that met inclusion criteria. Data were extracted and information was expressed according to data points as shown in Table 1.

**Table 1:** Article data, patient demographics, and risk factors. HTN - hypertension; PMHx- past medical history; POHx - past ocular history; Y - yes; N - no; RE - right eye; LE - left eye; NR - not reported; MD - myotonic dystrophy; IVT - iris vascular tufts; T1RF - type 1 respiratory failure; COPD - chronic obstructive pulmonary disease; T1DM - type 1 diabetes mellitus; T2DM - type 2 diabetes mellitus; CCF - congestive cardiac failure; IDA - iron deficiency anemia; IHD - ischemic heart disease; PVD - peripheral vascular disease; Ca - cancer; ECG - electrocardiogram; Tx - therapy; OA - osteoarthritis; Prev. - previous; PE - pulmonary embolism; CVA - cerebrovascular accident; VP - ventriculoperitoneal; Hx - history; AF - atrial fibrillation.

Author	Year	Sample	Age	Trauma	Blood dyscrasias	HTN	PMHx + medications	POHx + medications
Meades et al. [1]	1986	1	79	N	N	N	COPD, CCF	Glaucoma, cataracts
Fechner [2]	1958	1	42	N	N	N	Nil	Nil
Cobb [3]	1969	44	42 > 60, 2 < 60	NR	NR	NR	Diabetes mellitus, cardiovascular disease, and respiratory failure	One patient had bilateral disciform degeneration of the macula, three had retinal vein thrombosis, and one had blot hemorrhages
Cobb et al. [4]	1970	10	28-47	N	N	N	Five patients with MD and IVT; one with T1RF, one with left internal carotid thrombosis, and one with cylindroma of the salivary gland. Three patients were on quinine and one on steroids. The other five had bronchitis and epilepsy. Two patients were on quinine and one on steroids	Three patients had cataracts
Ooi et al. [5]	2015	1	86	N	Y (Warfarin)	Y	Warfarin - recurrent PE, hypothyroidism, HTN, CVA 30 years ago following VP shunt revision originally performed for pseudotumor cerebri	Nil
Dharmasena and Wallis [6]	2013	1	63	N	N	Y	HTN, Carotid artery disease. Bendroflumethiazide and aspirin. Cholesterol-lowering through diet	Nil
Williams et al. [8]	2018	14 (22 eyes)	58-82	N	N	NR	NR	Prev. hyphemas in 12 eyes, prev. glaucoma in seven eyes
Bakke and Drolsum [9]	2006	1	74	N	N	Y	COPD, Ischemic changes on ECG. 160 mg Aspirin. Beta-blocker	Idiopathic juxtafoveolar retinal telangiectasia bilaterally
Mason [10]	1979	60	38-68	N	N	N	NR	NR
Blanksma and Hooijmans [11]	1979	1	53	NR	N	N	Nil	Vascular sclerosis
Blanksma and Hooijmans [11]	1979	1	64	NR	N	N	Nil	Vascular sclerosis
Blanksma and Hooijmans [11]	1979	1	81	NR	N	N	COPD	Vascular sclerosis
Nuova et al. [12]	2020	1	74	N	N	Y	HTN - on antihypertensives	Nil
Krarup [13]	1977	1	36	N	N	N	Congenital heart disease - tricuspid atresia, atrial septal defect, truncus arteriosus, and persisting ductus arteriosus	Cyanotic retina with severe venous stasis. Myopia
Elgohary and Sheldrick [14]	2004	1	66	N	N	Y	HTN	Nil
Perry et al. [15]	1977	1	69	N	N	N	Emphysema	Nil
Ah-fat and Canning [16]	1993	1	55	N	N	N	Nil	Nil
Francis et al. [17]	1982	1	79	N	N	N	Heart failure, COPD	Cataracts and glaucoma – on pilocarpine and Timoptol
Francis et al. [17]	1982	1	59	N	N	N	Nephrectomy for Grawitz tumor, T2DM	Mild diabetic retinopathy
Francis et al. [17]	1982	1	83	N	N	N	Angina	LE – blind due to aphakic retinal detachment. RE– glaucoma receiving miotic and carbonic anhydrase inhibitor therapy, otherwise normal
Francis et al. [17]	1982	1	63	N	N	N	COPD	LE – amblyopia due to congenital exotropia
Straus et al. [18]	2005	1	80	N	N	Y	PVD with claudication, hypothyroidism, colon Ca - resected, prostate Ca - radiation Tx	Nuclear sclerotic cataract
Blanco et al. [19]	2019	1	71	N	N	N	Nil	Nil
Puri and Chan [20]	2001	1	59	N	N	Y	IHD, HTN	Nil
Sarmad et al. [21]	2018	1	61	N	NR	Y	HTN - on antihypertensives	Nil
Rosen and Lyons [22]	1969	1	73	Y	N	Y	HTN, chronic bronchitis	Strabismus on the opposite eye (RE)
Dahlmann and Benson [23]	2001	1	79	N	N	N	Nil	Nil
Coleman et al. [24]	1977	1	71	N	N	N	Nil	Nil
Jebaraj et al. [25]	2018	1	56	N	N	N	N	Family Hx of childhood glaucoma
Malik et al. [26]	2018	1	Late 50s	N	N	N	Prev. breast cancer. Primary biliary cirrhosis	Nil
Robinson et al. [27]	2008	1	51	N	N	N	OA. Naproxen, ibuprofen, calcium	Prev. spontaneous hyphemas treated conservatively
Robinson et al. [27]	2008	1	73	N	N	Y	HTN - amiloride-hydrochlorothiazide. AF, OA	Nil
Hagen and Williams [28]	1986	1	61	N	N	N	Nil	Nil
Welch [29]	1980	1	54	N	N	N	T2DM	Emmetropic, presbyopia, small choroidal naevus LE
Akram et al. [30]	2003	1	74	N	N	Y	HTN - atenolol	Nil
Bandello et al. [31]	1993	1	60	N	N	Y	HTN - amiloride and hydrochlorothiazide	Nil
Kang et al. [32]	2017	1	75	N	N	Y	Controlled HTN	Nil
Cota and Peckar [33]	1998	1	69	N	Y	Y	Hereditary hemorrhagic telangiectasia, HTN, IDA	Nil
Goyal et al. [34]	2010	1	69	N	N	N	Osteoporosis, hypothyroidism	Hypermetropia
Winnick et al. [35]	2003	1	75	N	N	N	Nil	Recurrent hyphemas LE. Multiple hemangiomas on the pupillary border bilaterally
Thomas et al. [36]	1988	1	75	N	N	N	Nil	Nil
Goetz and Cosgrave [37]	2016	1	60	N	N	N	Peptic ulcer, fibromyalgia	Nil
Goetz and Cosgrave [37]	2016	1	72	N	N	Y	T1DM, HTN, hyperlipidemia	Nil
Goetz and Cosgrave [37]	2016	1	53	N	N	N	Crohn's, vertigo	Treatment for glaucoma one week earlier
Goetz and Cosgrave [37]	2016	1	79	N	N	N	Hyperlipidemia	Non-neovascular age-related macular degeneration
Podolsky and Srinivasan [38]	1979	1	73	N	N	Y	HTN	Nil
Papastefanou et al. [39]	2016	1	70	NR	NR	NR	NR	NR

Results

This review compiled 38 articles based on the selection criteria including one case-control study, two cross-sectional studies, five case series, and 30 case reports, describing a total of 115 cases of iris vascular tufts. Since the last literature review in 2013, there have been 10 new articles reporting 26 new cases of IVTs. One article was a retrospective observational case series on 14 patients [8].

Patient Characteristics

Patients' ages ranged from 36 to 86 years with a mean of 65 years and median of 67 years. No sex or racial predisposition was noted. There was no case of prior intra-ocular trauma or surgery. Only one case of blood dyscrasia was reported with the patient being on warfarin [5]. Hypertension (48%), ischemic heart disease (18%), and diabetes (12%) were common among those with co-morbidities. Previous medical and ocular history is summarized in Table 1. The lesions appear as very small and nodular hemangioma on the iris and range from 15 to 150 microns in size [2,3]. They can be single or multiple and are most commonly bilateral [2,3]. The onset is usually in the sixth decade or older [3,4]. Meades et al. provided an electron microscopic description of IVT, indicating the nature of a true hamartoma of the iris stromal blood vessels [1].

Etiology and Other Associations

The exact cause of IVT remains unclear. Initially, these lesions were assumed to be congenital [2]. However, as there are no reported cases in children, these lesions are believed to be acquired [9]. Literature suggests a higher incidence of this lesion in those with systemic and other ocular conditions. Mason (1979) and Cobb et al. (1970) provided evidence that IVT can be associated with myotonic dystrophy and diabetes mellitus [3,4,10]. Mason’s investigation demonstrates an incidence of tufts in 6.7% of adult-onset diabetics and in 12.5% of patients with myotonic dystrophy. Tufts were not seen in 14 patients with juvenile-onset diabetes [10]. There appeared to be increased pancreatic B cell responsiveness in patients with myotonic dystrophy in response to glucose, thus causing high endogenous insulin levels. This study hypothesized that the higher levels of serum insulin might participate in the development of iris neovascularization by immunologic or other unknown mechanisms [10]. Cobb et al. (1969) hypothesized that tufts may proliferate in response to biochemical changes in the aqueous as in cataracts, diabetes, respiratory failure, and ocular hypotony [4]. Blanksma and Hooijmans (1979) also suggested that there seems to be a connection between the development of IVT and cardiovascular and pulmonary disease [11]. Systemically, they could also be associated with hypertension [12], congenital cyanotic heart disease [13], and congenital hemangiomatosis [6]. Nuova et al. (2020) presented a study of a patient with poorly controlled hypertension who presented with a spontaneous hyphema and hypertensive crisis [12]. Krarup (1977) reported a case of two years of bilateral IVT and recurrent microhyphema with the background of congenital cyanotic heart disease. The study summarized that a prolonged stasis with subsequent hypoxia of the iris tissue is a common factor in those conditions where a local or systemic disease is known to be present [13].

Cobb’s tufts have also presented simultaneously with other ocular manifestations. Elgohary and Sheldrick (2004) presented a case of spontaneous hyphema from IVT in the context of acute branch retinal vein occlusion. The authors concluded that hyphema from IVT may indicate a recent retinal vein occlusion and that their presence can be a risk factor for the development of hyphema during the acute stage of an ischemic retinal vein occlusion [14]. There has been a report of idiopathic juxtafoveolar retinal telangiectasis presenting concurrently with IVT, although no association could be identified [9]. Acute glaucoma has presented simultaneously with spontaneous hyphema secondary to IVT; however, their association cannot be accurately determined based on the lack of reports. Perry et al. (1977) felt the attack of glaucoma could be precipitated by the occurrence of the hyphema [15]. IVT may also present similar to amaurosis fugax as the tufts bleed occasionally and resolve rapidly. Care must be taken to avoid this misdiagnosis [16].

Signs and Symptoms

Cobb’s tufts usually remain asymptomatic [3,17]. They can rarely cause spontaneous hyphemas, for which the most common presenting complaint is a sudden blurring of vision [2,6,8,13,14,18-28]. Less common symptoms are eye pain, discomfort, and sudden loss of vision [5,12,29-31]. The symptoms usually resolve within 48 hours [2,5,23]. They may resolve as quickly as a few hours thus presenting with transient visual loss and mimicking amaurosis fugax [16]. The extent of visual disturbance is usually related to the degree of hyphema and patients may have a normal visual acuity (VA) [9] or deterioration to "light perception" [30]. In addition to hyphemas, patients will usually present with elevated IOP [11,30]. Persistently raised IOP risks damage to the optic nerve, thus requiring aggressive medical therapy. This usually settles down as the hyphema resolves [30]. Patients can even present with the only positive finding being raised IOP, leading to possible misdiagnosis of ocular hypertension or glaucoma [6]. In Ah-fat and Canning’s study (1994), a patient was misdiagnosed with "mild iritis" on two occasions. However, the patient noticed "blood in the eye," ultimately leading to a diagnosis of hyphema [16].

Investigations

The external ocular examination is usually normal. VA ranges from normal to light perception based on the degree of hyphema. Tonometry commonly reveals raised IOP when there is an observable hyphema [9]. If there was a hyphema, gonioscopy can reveal a trace of blood, otherwise, this part of the examination is usually normal [16]. There has been a case where the patient presented with acute angle-closure on gonioscopy, as well as a spontaneous hyphema secondary to IVT [15]. Fundoscopy is also largely unremarkable; however, Akram et al. provide a case demonstrating a hyperemic disc with a splinter hemorrhage and venous congestion. These changes did resolve within six weeks of topical therapy [30]. Similar retinal nerve fiber changes can also be seen if there is a simultaneous branch retinal vein occlusion [14]. A slit-lamp examination can demonstrate vascular abnormalities at the pupillary border and may reveal active bleeding or a blood clot; only a third of IVT were revealed by slit-lamp microscopy that was later demonstrated on iris fluorescein angiography (IFA) [22]. These changes can be subtle and further assessment with IFA is recommended to delineate the full extent of these lesions [22]. IVT appears as coils of tightly clustered, minute blood vessels at the pupillary margin and demonstrate early hyperfluorescence with late staining [22]. Photographic/videographic documentation is recommended to assess changes over time [19,29]. The fellow eye should also be imaged as IVT is most commonly bilateral [3,22]. Meades et al. (1986) provided the first electron microscopic description of an iris microhemangioma, indicating it to be a true hamartoma of the iris stromal blood vessels [1]. Histopathology illustrates endothelial cells surrounded by pericytes and loose connective tissue and electron microscopic evidence of normal cell thickness with no fenestrations [1]. Another valuable tool that has recently been reported is the anterior segment optical coherence tomography (OCT) of IVT. Its advantages include shorter acquisition time, no need for intravenous dye injection, and three-dimensional visualization of ocular tissue permitting segmental analysis of microvascular anatomy [32]. However, IFA remains the more commonly used mode of investigation supplements clinical examination. In addition to all the above, a full blood count, clotting screen, and urine analysis should be carried out, which are normal in the vast majority of cases. A fasting blood glucose and oral glucose tolerance test is also recommended due to the incidence of IVT in diabetics [3,10].

Differential Diagnosis

When trauma is excluded, clinicians should be vigilant of the following conditions. Uveal melanoma is the most vital differential diagnosis to exclude and this can be detected by IFA and serial examinations with photographic surveillance [1,6]. Hereditary hemorrhagic telangiectasis (HHT) is another differential that is a rare autosomal dominant disorder. It is characterized by multiple dilatations of capillaries and venules of skin, mucous membranes, and viscera that may cause bleeding. Conjunctival and eyelid involvement is common, which is not present in IVT [33]. Gonioscopy can be valuable in differentiating IVT from iris neovascularization [25]. Rubeosis can appear similar to IVT; however, the major morphological difference is IVT are elevated, not flat, and they are confined to the pupillary border [10]. Other differentials include inflamed iris vessels and iris hemangiomas [6].

Management

Conservative

Cobb’s tufts do not produce symptoms unless the patients develop a hyphema [17]. Most patients with IVT rarely develop bleeding and rarely re-bleed [21,27]. Studies have shown that most hyphemas resolve without requiring intervention, thus they are initially treated with time and bed rest [2,15,26,29,30]. There has been a case of spontaneous hyphema from IVT secondary to over-anticoagulation with warfarin and intravenous vitamin K was part of the management plan [5].

Raised IOP commonly presents with hyphemas and is a vital part of acute management. Prolonged elevation in IOP may lead to optic nerve damage. There have been many studies showing successful responses to acetazolamide and/or topical beta-blockers such as Timoptol [5,6,15,16,20,27,30,34]. Topical steroids and mydriatics/cycloplegics should also be considered and help to decrease light sensitivity and intraocular inflammation [5,8,20,26,27,29,34,35]. Conservative management is sufficient even if there is active bleeding or hyphema is recurrent or pronounced [34].

Laser Photocoagulation

There have been numerous studies documenting the treatment of hyphema caused by IVT with serial argon laser photocoagulation (ALP) [6,8,14,18,20,21,24,27,31,34-36]. Variable laser parameters are shown in literature, in terms of spot size, laser power, duration, and the number of spots targeted (Table 2). IFA prior to ALP or surgical treatment is necessary [6]. Dharmasena and Wallis (2013) employed a laser with neodymium-doped yttrium aluminum garnet (Nd:YAG) with YAG Pi settings with a good outcome and no recurrence within six months. The laser was aimed at an angle to reduce the risk of inadvertent macular burn. The parameters used were 500 um spot size, 100 mW power, 0.5 seconds, and eight confluent burns [6]. ALP was utilized for a single episode of active bleeding in four studies [21,27,28,36]. Williams et al. (2018) recently published a retrospective observational case series reporting a good outcome from the use of ALP in two cases of active bleeding despite medical therapy with topical steroids and atropine [8]. There have also been three reports of its use in single episodes of hyphema [20,24,31]. Only one of these five studies suffered a recurrence years later, which was managed conservatively [27]. Bandello et al.'s (1993) study demonstrated no further bleeding but showed angiographic evidence of new IVT, leading to two further treatments with ALP [31]. The rationale behind the use of ALP in single episodes of hyphema is unclear, especially since these have shown good outcomes with conservative management alone. Two cases have been documented of ALP use in recurrent hyphema prior to cataract surgery and have shown good outcomes [18,35]. The risk of bleeding from cataract surgery is unknown. Winnick et al. (2003) utilized parameters of 200 mW power for the 0.1-second duration on 50 spots of 200 um size [35]. It was theorized that closure of IVT would reduce the intra-operative risk of bleeding during surgery. Only minimal bleeding from the iris was observed and no further hyphema was documented in the postoperative period [35]. Interestingly, Goetz and Cosgrave (2015) report a case of cataract surgery without prior laser photocoagulation three months after a hyphema occurred in a patient with IVT. The procedure was carried out successfully without intra- or postoperative bleeding [37]. The follow-up periods post-procedure were variable and ranged from two to 228 months, with a mean of 42 and a median of 16 months (Table 2). Corneal complications include burns, persistent focal edema, and generalized edema with significant endothelial dysfunction. Strauss et al. (2005) employ clinicians to consider multiple treatment sessions at sufficient intervals to facilitate increased total laser energy requirements without promoting potential corneal complications [18]. Robinson et al. and Goyal et al. recommend that ALP is reserved for cases of hyphema recurrence or failure of medical therapy [27,34].

**Table 2:** Presentation, management, and suggestions from authors. IOP - intraocular pressure; N - no; Y - yes; NR - not reported; IFA - iris fluorescein angiography; FA - fluorescein angiography; IVT - iris vascular tufts; N/A - not applicable; ALP - argon laser photocoagulation; MD - myotonic dystrophy; LE - left eye; RE - right eye; B-blockers - beta-blockers; HHT - hereditary hemorrhagic telangiectasia; OA - open angle; IV - intravenous; OCTA - optical coherence tomography angiography; Anti-HTN - antihypertensives; Nd:YAG - neodymium-doped yttrium aluminum garnet; s - second; Approx. - approximately; Prev. - previous; VA - visual acuity; mW - milliwatt; μm - micrometer; nm - nanometer.

Author	Year	Raised IOP	Hyphema	Duration of symptoms	Management of condition	Recurrence	Suggestions from author
Fechner [2]	1958	N	Y (micro)	Seven hours blurred vision and eye pain	Placebo. Observation. Hyphema resolved within 24 hours	N	The iris vascular pattern with micro-aneurysms at pupillary margins is presumed to be congenital
Rosen and Lyons [22]	1969	NR	Y (micro)	24 hours blurred vision	Bed rest	N	IFA showed more extensive vascular lesions at the pupillary border of both eyes than clinical findings indicated. These are most likely congenital
Cobb [3]	1969	N	N	Asymptomatic	Nil	N/A	The first paper to provide a detailed description of tufts and how it differs from rubeosis iridis. It also provides evidence of IVT's possible association with systemic conditions like diabetes, vascular, or respiratory disease
Cobb et al. [4]	1970	N	N	Asymptomatic	Nil	N/A	IVT can be associated with MD. Hypothesized that tufts may proliferate in response to biochemical changes in the aqueous as in cataracts, diabetes, respiratory failure, and ocular hypotony
Perry et al. [15]	1977	Y	Y (macro)	24-36 hours	Topical pilocarpine, oral acetazolamide and glycerol	N	Usually bilateral, male predominance, 6th-7th decade, associated with MD, respiratory disease, and diabetes
Coleman et al. [24]	1977	N	Y (micro)	‘Smoky’ vision. No duration	ALP	N - within two months follow up	IFA delineated the IVT. ALP eradicated the tufts that bled. Most of the patients with IVT have no systemic disease but they have been seen in diabetes and MD
Krarup [13]	1977	N	Y (micro - bilaterally)	Few days of "misty" vision	Nil	Y - in both eyes, multiple times over two years	A prolonged stasis with subsequent hypoxia of the iris tissue is a common factor in those conditions where a local or systemic disease is known to be present
Mason [10]	1979	N	N	Asymptomatic	Nil	N	IVT was associated with systemic conditions like MD and diabetes
Blanksma and Hooijmans [11]	1979	N	Y (macro)	Diminished vision for "short period"	Observation. Resolved within two days	N - within one year	These vascular lesions can be caused by cardiovascular dis­eases and by elevated venous pressure caused by intrathoracic processes
Blanksma and Hooijmans [11]	1979	Y	Y (macro)	Few weeks temporary decrease in VA	Bed rest. Diclofenamide. Resolved within two days.	N - within four years	
Blanksma and Hooijmans [11]	1979	N	Y (macro)	Sudden decrease in vision after getting out of bed	Observation. Resolved within three days	N - within 18 months	
Podolsky and Srinivasan [38]	1979	N	Y (macro)	Sudden painless onset of "red spot"	Topical homatropine and dexamethasone. Hyphema cleared in three days	N - within four years	IVT is usually asymptomatic. They can give rise to spontaneous hyphemas. ALP can be reserved for recurrent hyphema
Welch [29]	1980	N	Y (macro)	One day. Unilateral eye pain	Homatropine, bed rest, and eye padding. Resolved after one day	N	Nil
Francis et al. [17]	1982	N	N	Asymptomatic	Nil	N	
Francis et al. [17]	1982	N	N	Asymptomatic	Nil	N	
Francis et al. [17]	1982	N	N	Asymptomatic	Nil	N	
Francis et al. [17]	1982	Y	Y (micro)	Sudden clouding of vision	Bed rest and acetazolamide	N - within 12 months follow up	IVT are not uncommon lesions, and although they are usually asymptomatic, they now form an important part of the differential diagnosis of spontaneous hyphema, whether unilateral or bilateral. The vast majority will probably never need any intervention
Meades et al. [1]	1986	N/A	N/A	N/A	N/A	N/A	The first electron microscopic description of an IVT, indicating it to be a true hamartoma of the iris stromal blood vessels
Hagen and Williams [28]	1986	Y	Y (macro)	Eight hours blurred vision and "colored part of the eye covered in blood"	Topical timolol, cyclopentolate, and acetazolamide. ALP - four 200μmspots (240 mW for 0.2 s). Hyphema cleared in four days	N - within two months follow up	Case of efficient and successful use of ALP to treat bleeding IVT without complications
Thomas et al. [36]	1988	Y	Y (micro)	Two hours. Sudden loss of vision and eye pain	ALP - two shots, 200 μmspot size, 0.3-0.4 mW power, and 0.2 s duration. The pupil was dilated with phenylephrine and tropicamide drops. One drop of timolol and eye was patched	N	The discovery of an IVT per se is not an indication for treatment, but treatment in the case of active bleeding is probably justified
Ah-fat and Canning [16]	1993	Y	Y (micro)	Two hours. Sudden loss of vision	Topical acetazolamide and b-blockers. Observation	Y - four episodes over the last six months	May mimic amaurosis fugax. Gonioscopy may be useful in revealing a small resolving hyphema. Unnecessary investigations and treatment of the carotid circulation may thus be avoided
Bandello et al. [31]	1993	N	Y (Micro)	One day. Sudden vision loss	Topical tropicamide. ALP: dye-yellow (577 nm), 100 mW, 250μm​​​​​​ spot size, 2 s duration, no. of spots = 10	No recurrence of hyphema within 12 months. Recurrent of IVT, which underwent repeat laser therapy twice	Control fluoroiridographic follow-up is very important for patients who have undergone ALP for vascular tufts to evaluate the reappearance or formation of lesions over time and to prevent recurring hyphema
Cota and Peckar [33]	1998	N	Y (LE macro, RE micro)	Sudden onset blurred vision	Bed rest. Resolved spontaneously within two weeks	N	Iris vascular malformations may over in HHT and cause spontaneous hyphema. It should be considered as a differential in those with IVT or spontaneous hyphema
Puri and Chan [20]	2001	Y	Y (macro)	One day. Blurred vision	Topical prednisolone and Timoptol. Angiography-guided ALP (patient advised for this procedure). Gradual resolution and normalization of IOP over days 1, 5, and 7	N	Cobb’s tufts are a rare cause of spontaneous hyphema in the elderly. Raised IOP is a common finding in hyphema and should be treated appropriately. Hematological and coagulation profiles should be performed in patients with spontaneous atraumatic hyphema. If in doubt, IFA can help provide vital clues
Dahlmann and Benson [23]	2001	Y	Y (micro)	<24 hours. Sudden onset ocular pain, redness, and blurred vision	Topical atropine, betamethasone, and carteolol	N	These lesions can be single, multiple, or bilateral. IFA demonstrates leakage from these lesions. Origin unknown. Most have no systemic disease. Recurrent episodes of spontaneous hyphema, ALP can be used
Winnick et al. [35]	2003	N	Y (macro)	NR	1st episode: topical homatropine and prednisolone 0.1 s duration. 50 spots. 2nd episode: ALP. 200 μm spot size, 200 mW power	Y - for two years	Consider ALP to treat IVT before surgery to decrease the possibility of intraoperative and postoperative complications of uncontrolled hemorrhaging
Akram et al. [30]	2003	Y	Y (macro)	Six hours. Sudden vision loss	Observation. Acetazolamide, topical b-blockers, and fluorometholone for six weeks	N	There is not much literature dealing with the treatment of such lesions, and clearly, the rarity of these patients limits the development of a management protocol
Elgohary and Sheldrick [14]	2004	N	Y (micro)	One day. Blurred vision	Aspirin 75 mg. Dietician for hypercholesterolemia. Six months follow-up - VA improved, ongoing slight blurred vision	Y - nine months later, treated with scatter laser to the ischemic retina	Spontaneous hyphema from IVT may indicate a recent onset of a retinal venous occlusion. Their presence can be a risk factor for the development of hyphema during the acute stage of an ischemic retinal venous occlusion. Hemodynamic changes may increase intravascular pressure of tufts and cause hyphema
Straus et al. [18]	2005	N	N	One to two days. Transient blurring of vision	Serial ALP to iris tufts. 260-270 mW, 50 μmspot size, 0.1s total energy. Prev. episodes treated with time	Y - for 20 years, approx. every three months	No pupillary function damage. The treated eye has cleared condition at 20 months follow up
Bakke and Drolsum [9]	2006	N	Y (macro)	Asymptomatic	Topical steroids. Aspirin reduced. Observation	N	IFA can show further IVT. ALP is recommended prior to intraocular surgery. Acquired condition suggesting specific risk factors occur. Excision may be possible. Bipolar diathermy has also been used
Robinson et al. [27]	2008	Y	Y (micro)	One day "hazy" vision	Topical steroid, cycloplegic, and b-blocker	N - within three years follow up	IVT must be included in the differential diagnosis of spontaneous hyphema. IFA may be helpful. Observation is often warranted, as bleeding is uncommon and recurrent episodes are rare. ALP may not prevent rebleeding
Robinson et al. [27]	2008	N	Y (micro)	One day "foggy" vision	ALP	Y - Two further recurrence within 15 years. The patient refused laser treatment	
Goyal et al. [34]	2010	Y	Y (micro)	One day. Sudden blurred vision	Steroid, cycloplegic and hypotensive drops, and oral acetazolamide	N	Conservative management is sufficient even if there is active bleeding or hyphema is recurrent or pronounced. Although none of the reports noted any adverse effects with ALP, IVT rarely needs intervention
Dharmasena and Wallis [6]	2013	Y	Y (micro - bilaterally)	One to two days. Transient blurring of vision	ALP with Nd:YAG with YAG Pi settings, aimed at an angle to reduce the risk of inadvertent macular burn. 500μmspot size, 100 mW power, 0.5 s, eight confluent burns. Acetazolamide and antiglaucoma medication	N - within six months follow up	YAG Pi settings, angled laser beam. Worth bearing in mind with intermittent secondary OA glaucoma
Ooi et al. [5]	2015	Y	Y (macro)	Eight hours. Sudden, persisting vision loss	Combination of prednisolone, atropine, brinzolamide, timolol, latanoprost, and brimonidine. Vitamin K IV to normalize INR. Condition improved within 48 hours	N	The possibility of over-anticoagulation should always be considered. Topical and systemic steroids can be used to induce IVT regression or hasten spontaneous shrinkage. Conservative management of IVT in the initial instance is more than appropriate
Papastefanou et al. [39]	2016	NR	NR	NR	NR	NR	NR
Goetz and Cosgrave [37]	2016	Y	Y (macro)	Four hours. Sudden onset blurred vision	Topical dexamethasone and IOP lowering agents. Full recovery	N	Important to carefully examine all eyes. IVT is more numerous than clinically apparent. IFA can prove invaluable. The majority of hyphemas can be treated conservatively
Goetz and Cosgrave [37]	2016	N	Y (macro)	Blurred vision on awakening	Topical steroid and cycloplegic. Full recovery	N	
Goetz and Cosgrave [37]	2016	Y	Y (macro)	24 hours. Sudden blurred vision	Topical brinzolamide, timolol, acetazolamide, and dexamethasone. Full recovery	N	
Goetz and Cosgrave [37]	2016	Y	Y (macro)	Five hours. Blurred vision	Topical dexamethasone, cycloplegic, and apraclonidine. Full recovery	Y- once a month later	Reports case of phacoemulsification on the background of IVT. Uncomplicated procedure. ALP was not required prior to the procedure
Kang et al. [32]	2017	N	N	Asymptomatic	Observation	N	This is the first report of OCTA of IVT. Although FA has traditionally been effective in highlighting iris vascular lesions, the non-invasive nature and depth-localizing strengths of OCTA are appealing
Sarmad et al. [21]	2018	Y	Y (macro)	One day. Blurred vision and discomfort	ALP. ARI 532nm, two burns, 50 μm​​​​​​ spot size, 0.1 s, 400 mW. Dorzolamide, timolol, and prednisolone	N - within five years of follow up	The literature suggests that pre-treatment is indicated if intra-ocular surgery is to be conducted. Only two burns are required for excellent treatment of this condition at five years
Williams et al. [8]	2018	Y	Y (two patients - micro)	Blurred vision in 13 eyes	Observation in 14 cases. In seven patients and had topical anti-HTN. Topical steroids or atropine in four cases. ALP in two cases resulting in complete hemostasis	Y - (one case which was followed up for 85 months)	Observation in those without ongoing signs or symptoms. Topical steroids and atropine could be beneficial if hyphema persists. Topical anti-HTN for raised IOP. ALP for persisting bleeding or recurrence
Jebaraj et al. [25]	2018	N	Y (micro)	Blurred vision. No duration	Bed rest. Topical prednisolone and cyclopentolate	Y	Gonioscopy to differentiate IVT from iris neovascularization. Topical steroids and mydriatics for hemostasis can be used. IOP monitoring and treatment is an important component
Malik et al. [26]	2018	Y	Y (micro)	Blurred vision. No duration	Topical corticosteroid, cycloplegic, and aqueous suppressant	N - within four months follow up	Observe the patient in the first instance. Reserve ALP for hyphema recurrence
Blanco et al. [19]	2019	N	Y (micro)	Two days. Blurred vision	Repeated digital compression over the superior eyelid. The bleeding stopped the next day	N - within nine months follow up	Usually, only require medical treatment to either treat or avoid IOP spikes. Performing digital compression repeatedly could help achieve hemostasis
Nuova et al. [12]	2020	N	Y (micro)	Acute visual deterioration	Topical prednisolone and cyclopentolate. Resolved within 15 days	N - within 24 months follow up	Hypertensive crisis in the patient presented here triggered the occurrence of the hyphema. Ultrasound microscopy could be utilized in order to rule out neoplasms of the iris and ciliary body. Blood pressure control is important to avoid complications

Surgery

Iridectomy offers the benefits of no recurrence and clinically excludes a malignant lesion. Good outcomes have been shown from the procedure. The literature recommends that this should be considered in patients who are still symptomatic despite laser treatment or in case of suspicion regarding a malignant lesion [6].

Discussion

Cobb’s tufts are coils of tightly clustered, minute blood vessels at the iris pupillary margin [22]. The etiology and pathogenesis of this condition are largely unknown. To date, there is no literature based on these aspects. As there are no reported cases in children, these tufts are believed to be acquired [9]. Moreover, there is a higher incidence of Cobb’s tufts in patients with systemic conditions such as myotonic dystrophy and diabetes mellitus [3,4,10]. IVT also seems to occur more in patients with cardiovascular, respiratory disease, and hypertension [11,12]. There are also single reports of IVT presenting as spontaneous hyphemas in patients with congenital heart disease, congenital hemangiomatosis, and hereditary hemorrhagic telangiectasia, respectively [6,13,33]. Clinicians should also be aware of possible ocular associations. Interestingly, these lesions have presented as hyphemas in the context of acute branch retinal vein occlusion, idiopathic juxtafoveolar retinal telangiectasis, and acute glaucoma [9,14,15]. The authors would recommend considering the possibility of the aforementioned systemic and ocular associations when assessing patients with spontaneous hyphemas and/or IVT.

Spontaneous hyphema can be a rare sequela of IVT. The most common presenting complaint is sudden blurring of vision [2,6,8,13,14,18-28,38]. However, they can also present as eye pain, discomfort, sudden loss/deterioration of vision, or "blood in the eye" [5,12,29-31]. Symptoms usually resolve within 48 hours [2,5,23]. Hyphemas are commonly associated with raised IOP and this can be transient leading to a misdiagnosis of ocular hypertension or glaucoma [6]. Examination generally consists of external ocular examination, VA assessment, tonometry, gonioscopy, fundoscopy, and slit-lamp examination. IVT can be easily missed on clinical examination alone and the authors would recommend supplementing the aforementioned modes of examination with IFA.

IFA is helpful to investigate the full extent of these lesions. They are commonly found to be bilateral, more numerous, and demonstrate early hyperfluorescence with late staining [22]. IFA can be combined with serial examinations to exclude iris melanoma. The authors would recommend considering differential diagnoses such as rubeosis, iris neovascularization, iris hemangiomas, HHT, and inflamed iris vessels [6,10,25,33]. However, there is a weak level of evidence surrounding differential diagnosis for IVT.

Prior to treatment, it is commonly reported that IFA is performed [6]. The majority of spontaneous hyphemas secondary to Cobb’s tufts can be treated conservatively. Conservative modalities include bed rest and topical therapy including IOP-reducing drugs such as beta-blockers, steroids, and mydriatics/cycloplegics [2,15,26,29,30]. Literature has shown the successful use of ALP in active bleeding, single episodes of bleeding, and recurrent hyphemas [6,8,14,18,20,21,24,27,31,34-36]. The indication for laser treatment in single episodes of bleeding remains unclear due to the very good outcomes of conservative management. Therefore, we would recommend that ALP is reserved for cases of hyphema recurrence or failure of medical therapy.

The benefit of ALP prior to intraocular surgery is unknown. There have been two studies of ALP prior to cataract surgery, one of which resulted in minimal bleeding intra-operatively [18,35]. On the other hand, Goetz and Cosgrave (2016) reported a case of cataract surgery in a patient with IVT and reported no complications or bleeding [37]. Iridectomy can be considered in patients who are still symptomatic despite ALP or if there is suspicion of malignancy.

Dharmasena and Wallis provided us with the last literature review on this topic in 2013, there have been 10 new articles reporting 26 new cases of IVT. One issue is the majority of these studies are case reports and consist of a very small sample size (one to four). Some studies also add further evidence of known knowledge regarding Cobb’s tufts but lack any new breakthroughs [12,21,25,26,39]. These articles reiterate that conservative management is recommended initially, the importance of controlling IOP and blood pressure and that ALP should be reserved for recurrent hyphemas. Blanco et al. (2019) provided rare video documentation of the course of this condition. They utilized repeat digital compression of the superior eyelid as part of conservative management [19]. The authors feel this is unlikely to be of many benefits since the majority of such hyphemas settle with bed rest and time. Another study highlighted the risk of anticoagulation therapy as a predisposing factor for spontaneous hyphema and this should factor into management considerations [5].

Recent updates in literature, such as Williams et al.'s (2018) observational study of 14 patients (22 eyes), further confirm that IFA is a diagnostic modality and that ALP should be reserved for recurrence [8]. This was the second recent article to mention optical coherence tomography angiography (OCTA) as an alternative to IFA [32]. The non-invasive nature and depth-localizing strengths of this new development are appealing but require further studies before it would surpass IFA. Another recent study by Goetz and Cosgrave makes us rethink the use of ALP prior to intraocular surgery in eyes containing IVT [37]. The authors feel that further studies are required surrounding ALP prior to intraocular surgery in the context of IVT as conflicting results have been shown in the very few studies available.

Limitations

The sum of this systematic review is limited by the published literature due to the relative scarcity of the condition. Most papers are case discussions or small number case series. There has been limited research into etiology and much remains to be investigated.

## Conclusions

Cobb’s tufts are benign true hamartomas of the iris stromal vessels at the pupillary border. They cause no symptoms unless a hyphema develops, which most commonly presents as blurred vision. A single episode of bleeding is rare and re-bleeding is also a rare occurrence. Recent literature provides further evidence to support conservative treatment of a spontaneous hyphema in the first instance, given the reports of great resolution rates and low recurrence. Laser photocoagulation has a role in cases of hyphema recurrence despite medical therapy. A large array of settings is employed resulting in uncertainty regarding the optimal settings for laser photocoagulation. Current literature largely consists of case reports/series making for weak evidence regarding etiology, laser photocoagulation, and surgical treatment. OCTA has recently emerged as an alternative imaging modality for these lesions and demonstrates favorable initial advantages over IFA. However, larger prospective studies are needed to research this imaging modality, in addition to the optimal management strategy to use with laser photocoagulation and iridectomy for these lesions.
